# The Sociability Argument for the Burqa Ban: A Qualified Defence

**DOI:** 10.1007/s11572-021-09622-4

**Published:** 2021-11-09

**Authors:** Bouke de Vries

**Affiliations:** Department of Historical, Philosophical and Religious Studies, Umeå University, Umeå, Sweden

**Keywords:** Burqas, Niqabs, Face-coverings, Burqa Ban, Islam, Living together, Facemasks, Sociability, Loneliness

## Abstract

Over the past decade, countries such as France, Belgium, Denmark, Austria, Latvia, and Bulgaria have banned face-coverings from public spaces. These bans are popularly known as ‘burqa bans’ as they seem to have been drafted with the aim of preventing people from wearing burqas and niqabs specifically. The scholarly response to these bans has been overwhelmingly negative, with several lawyers and philosophers arguing that they violate the human right to freedom of religion. While this article shares some of the concerns that have been raised, it argues that banning face-coverings in public is morally justified under certain conditions with the exception of facemasks that are necessary for the containment of infectious diseases, such as COVID-19. The reason for this is that those who publicly cover their face make it very difficult for other members of society to socially interact with them, especially for those who are deaf or hard-of-hearing, which is problematic in an age where many people are chronically lonely or at risk of becoming chronically lonely. As such, this article can be understood as a more elaborate, and arguably more sophisticated, defence of the justification that France offered for its face-covering ban before the European Court of Human Rights, namely that covering one’s face undermines the conditions for ‘living together’.

## Introduction

Over the past decade, countries such as France, Belgium, Denmark, Austria, Latvia, and Bulgaria have banned face-coverings from public spaces.^1^ These bans are popularly known as ‘burqa bans’ as they seem to have been drafted with the aim of preventing people from wearing burqas and niqabs specifically. The scholarly response to these bans has been overwhelmingly negative, with several lawyers and philosophers arguing that they violate the human right to freedom of religion.^2^ While this article shares some of the concerns that have been raised, it argues that banning face-coverings in public is morally justified under certain conditions with the exception of facemasks that are necessary for the containment of infectious diseases, such as COVID-19. The reason it offers is *not* that wearing burqas or niqabs is inherently degrading to women, or that it reinforces the oppression of Muslim women as some feminists have argued.^3^ Even when none of these things are true, I will argue that bans on public face-covering remain morally justified on grounds that those who publicly cover their face make it very difficult for other members of society to socially interact with them, especially for those who are deaf or hard-of-hearing. As I show, this is problematic because if a large proportion of the population were to dress in this way, the opportunities for people to have micro-moments of connection with strangers would become unduly small, which is problematic in turn because sharing such moments is an important safeguard against chronic loneliness and its harmful health effects. As such, this article can be understood as a more elaborate, and arguably more sophisticated, defence of the justification that France offered for its face-covering ban before the European Court of Human Rights (ECtHR), namely that covering one’s face undermines the conditions for ‘living together’.^4^ However, I end by arguing that, in the same way that some countries exempt citizens from compulsory military service if they are prepared to do an alternative civilian service,^5^ those who wish to cover their face in public should be allowed to do so if they are willing to performing specific services that help to protect fellow members of society from chronic loneliness.

## The Proliferation of Face-Covering Bans in Europe

In July 2010, France was the first European country to pass a bill that banned face-coverings from public spaces.^6^ The relevant law (‘Prohibiting the concealment of one’s face in public places’) was subsequently approved by the French senate in September that year, before coming into force in April 2011. Under its Sects. 1 and 2, ‘no one may, in public places, wear clothing that is designed to conceal the face’, whereby public spaces are understood to compromise the ‘public highway and any places open to the public or assigned to a public service’.^7^ The only exceptions are cases where face-covering clothing ‘is prescribed or authorized by primary or secondary legislation, if it is justified for health or occupational reasons, or if it is worn in the context of sports, festivities or artistic or traditional events’.^8^ Failure to comply with these restrictions is punishable by a fine of up to 150 euros and/or an obligation to follow a citizenship course per Sect. 3, whereas forcing another person to ‘conceal their face, by threat, duress, coercion, abuse of authority or of office, on account of their gender’ is punishable by a one year prison sentence and a fine of 30,000 euros that is raised to 60,000 euros if the coerced individual is a minor per Sect. 4.^9^

According to the explanatory memorandum to the law,^10^ the reason why these restrictions were deemed necessary is that ‘the voluntary and systematic concealment of the face is […] quite simply incompatible with the fundamental requirements of living together in French society’ by falling short of the ‘minimum requirement of civility that is necessary for social interaction’. To make sense of this statement, one needs to understand the political context in which the law was implemented. It is no secret that its implementation was motivated by a widespread desire on the part of French legislators to ban two types of Islamic veil specifically if not exclusively, namely the burqa (which covers all of the face) and the niqab (which leaves an opening for the eyes).^11^ One clue for this is that back in 2009, then President Nicolas Sarkozy had publicly said that ‘the burqa is not welcome in France’^12^ and soon thereafter established a commission consisting of 32 lawmakers to study the practice of wearing the full veil and to explore ways of restricting this practice.^13^ Another clue is that the parliamentarian debate preceding the law’s enactment focused on the banning of the burqa and niqab specifically.^14^

Now, as soon as the law came into effect, its legality was challenged by an anonymous French citizen who lodged an application with the ECtHR against the French state.^15^ According to the applicant, who claimed to wear the full veil for religious, cultural, and personal reasons, the ban on public face-covering violated several of her human rights under the European Convention of Human Rights (ECHR). These included Article 3 (the right against inhuman or degrading treatment), Article 8 (the right to respect for private and family life), Article 9 (the right to respect for freedom of thought, conscience, and religion), Article 10 (the right to freedom of expression), and Article 11 (the right to freedom of association), taken both separately and together with Article 14 (the prohibition of discrimination). After the case had been forwarded to the Grand Chamber by the Fifth Section of the Court, a verdict was delivered on 1 July 2014. Rejecting several of the applicant’s complaints as inadmissible, the Court considered the central issue to be whether France’s face-covering ban violated Articles 8 and 9 of the ECHR. Whereas Article 8 Sect. 1 recognises the right of everyone ‘to respect for his private and family life, his home and his correspondence’, Article 8 Sect. 2 allows this right to be limited if, and only if, this ‘is in accordance with the law and is necessary in a democratic society in the interests of national security, public safety or the economic well-being of the country, for the prevention of disorder or crime, for the protection of health or morals, or for the protection of the rights and freedoms of others’. Article 9 has a similar structure with its first section recognising everyone’s right to ‘freedom of thought, conscience and religion’, including ‘freedom to change his religion or belief and freedom, either alone or in community with others and in public or private to manifest his religion or belief, in worship, teaching, practice and observance’ and its second section allowing this right to be limited if, and only if, the limitations imposed are ‘prescribed by law and are necessary in a democratic society in the interests of public safety, for the protection of public order, health or morals, or for the protection of the rights and freedoms of others’. Granting a wide margin of appreciation to France,^16^ the Court ruled that, while its face-covering ban interfered with both Articles, the limitations imposed on these rights were justified by their respective limitation clauses, noting that the ban ‘can be regarded as proportionate to the aim pursued, namely the preservation of the conditions of “living together” as an element of the “protection of the rights and freedoms of others”’.^17^

As already alluded to, France was the first European country but certainly not the last to introduce a public face-covering ban. Belgium followed in 2011 when its Chamber of Representatives adopted a law ‘to prohibit the wearing of any clothing entirely or substantially concealing the face’, which was made punishable with ‘a fine of between fifteen and twenty-five euros and imprisonment of between one and seven days, or only one of those sanctions’.^18^ The country also followed in France’s footsteps in having to defend its face-covering ban before the ECtHR after its legality had been challenged by one Belgium national and one Moroccan national who both wore the niqab in public. As in the *S.A.S* case, however, the Court ruled that there was no violation of Articles 8, 9, and 14 of the European Convention and upheld the ban unanimously.^19^ Between 2015 and 2021, several more European countries went on to ban face-coverings from (certain) public spaces. Whereas Denmark, Austria, Latvia, and Bulgariaintroduced bans covering *all* public spaces like the ones France and Belgium had implemented,^20^ Norway and the Netherlands introduced bans that apply to specific public places only, which include nurseries, schools, and universities in the case of Norway^21^ and public buildings, hospitals, educational institutions, and public transport in the case of the Netherlands.^22^

## The Sociability Argument for Face-Covering Bans

The ECtHR’s verdict in *S.A.S. v France*, which we have just seen set the stage for a proliferation of public face-covering bans across Europe, has not been without controversy. In their joint dissenting opinion, judges Angelika Nussberger and Helena Jäderblom rejected the notion that the value of living together can justify bans on public face-covering and referred to the concept of living together as ‘far-fetched and vague’,^23^ a view shared by philosopher Sune Lægaard^24^ and legal scholar Eva Brems.^25^ In the remainder of this article, my aim is to defend the French state against these criticisms. To be more precise, I will argue that there is a particular aspect of living together that is important enough to justify banning face-coverings from public spaces with the exception of facemasks that are necessary for the containment of infectious diseases such as COVID-19 (which I take to be justified on grounds of public health).

The aspect of living together that I have in mind consists of a particular form of human sociability, namely amicable interactions with strangers that occur when we exchange smiles or greetings with those we do not know, or when we engage in small talk with them in a variety of publicly accessible settings such as buses, trains, grocery stores, parks, and public squares. With Barbara Fredrickson, I will refer to such interactions as ‘micro-moments of connection’.^26^ While their short-lived nature might suggest that micro-moments of connection are trivial, the opposite is true as Fredrickson, who is a leading researcher on emotions and relationships, has shown in *Love 2.0: Finding Happiness and Health in Moments of Connection*.^27^ Drawing upon decades-long research from psychology and the neurosciences, she finds that ‘whenever two or more people—even strangers—connect over a shared positive emotion, be it mild or strong’, this is accompanied by a synchronisation between their biochemistry and behaviours and by expressions of mutual care that have a significant positive impact upon people’s well-being and quality of life, no matter how fleeting such moments might be.^28^

It is the value of micro-moments of connection with strangers and its ability to protect people from chronic loneliness in particular that I believe justifies bans on public face-covering within contemporary European societies (on which I focus here). In premise-form, this argument might be formulated as follows:

### The Sociability Argument for Face-Covering Bans

1. If certain behaviours cause significant harm to the health of others if enough people engage in them, then it is morally permissible for states to restrict the relevant behaviours *regardless* of how many people actually engage in them, provided this does not impose unreasonable costs or burdens.
2. If a large proportion of individuals were to cover their face in public, this would make it much more difficult, and often very difficult overall, for people to share micro-moments of connection with strangers, especially for those who are deaf or hard-of-hearing.
3. In contemporary European societies, micro-moments of connection with strangers are critical for protecting certain groups of people from chronic loneliness.
4. Chronic loneliness takes a heavy toll of people’s mental and physical health.
  Therefore,
5. It is morally permissible for contemporary European states to ban public face-covering as long as this does not impose unreasonable costs or burdens.
6. Banning public face-covering does not impose unreasonable costs or burdens when individuals are able to receive exemptions from such bans in exchange for delivering certain services that help to protect fellow members of society from chronic loneliness.

Therefore,

In contemporary European societies, banning public face-covering is morally justified under certain conditions.

Given that this argument is valid, the truth of its premises must entail the truth of its conclusion. My aim in the remainder of this article is to suggest that its premises are indeed true. To do so, I will start by vindicating premises 1–4, which I will address in reverse order.

### Premise 4

As a preface to premise 4, I should mention that virtually everyone feels lonely during certain moments of their lives (e.g. in the wake of a relocation or a romantic break-up) understood as the negative feelings (e.g. sadness, despair) that we experience as a result of mismatches between, on the one hand, the number or kinds of relationships that we want and, on the other, the relationships that we have, or simply believe to have.^29^ Since we manage to overcome such feelings in most cases, their presence is not a major concern in and of itself.^30^ However, once our loneliness becomes persistent or chronic, serious problems start to arise. To see this, it should be noted that chronic loneliness has been found to contribute to various mental and physical health problems, including depression;^31^ dementia;^32^ alcoholism;^33^ increased mortality;^34^ and poor physical health;^35^ with some experts arguing that its effects upon people’s health can be compared to those of smoking 15 cigarettes a day.^36^

### Premise 3

The easiest way of demonstrating that moments of micro-connection with strangers are critical for protecting certain groups from chronic loneliness within contemporary European societies is to point out that such moments have been found to offer protection from loneliness^37^ and that, for some people within these societies, other types of social interaction are (largely) unavailable, or simply incapable of offering sufficient protection.^38^ One reason for this might be that they have few, if any, friends and relatives to interact with, which is a problem that is especially common among older adults. Due mostly to the death of friends, siblings, and romantic partners, studies have found that the social networks of those aged 85 and above are on average approximately half the size of the social networks of 70–84-year-olds,^39^ which is reflected in self-reports of loneliness. According to surveys from Europe and North America, circa 20 to 35 percent of adults between the ages of 65 and 79 say that they are often lonely, a figure that rises to 40 to 50 percent among those aged 80 and above.^40^ Yet even when people have (frequent) contact with friends and relatives, these relationships might still fail to protect them from loneliness when their quality is low, as when they find themselves in a dysfunctional romantic relationship. For just as having a small number of relationships has been shown to leave individuals at risk of becoming or remaining lonely,^41^ so having low-quality relationships has been shown to do so.^42^ It is in these two circumstances, i.e. in ones where social interactions with friends and relatives are (largely) unavailable and ones where they are available but where their quality is poor that micro-moments of connection with strangers are especially important safeguards against (chronic) loneliness.

### Premise 2

The premise that if a large proportion of individuals were to cover their face in public spaces, this would make it much more difficult, and often very difficult overall, for people to share micro-moments of connection with strangers is supported by two observations. The first is that public spaces are the domain where micro-moments of connection between strangers primarily take place. Since I take this to be uncontroversial, I will not say more about it here.

The second observation is that covering all of one’s face, or all of one’s face but one’s eyes, creates a significant barrier to communication with strangers. This barrier is especially large for those who are deaf or hard-of-hearing, who are estimated to compromise five percent of the world population.^43^ For this group, being unable to see other people’s facial expressions and read their lips often deprives them of much, if not most, of the information they need to communicate effectively,^44^ which explains, among other things, why many deaf people are reporting that the use of mouth-covering masks during the current COVID-19 pandemic is hampering their ability to communicate with fellow members of society, as well as why some of them are campaigning for the use of transparent masks that leave the mouth visible.^45^ For example, Fizz Izagaren, a pediatric doctor from the UK who has been deaf since the age of two, describes how ‘when someone is wearing a [non-transparent] face mask I’ve lost the ability to lip read and I’ve lost facial expressions—I have lost the key things that make a sentence’.^46^

But it is not only those with hearing disabilities who may find it challenging to communicate with individuals whose faces are covered. Psychological research has shown that human facial displays facilitate effective communication by enabling us to signal to our interlocutor in which direction we want the conversation to go,^47^ which are cues that are missing when people’s faces are covered. In addition to this, some studies have shown that we tend to misjudge the strength of people’s emotions when their faces are covered. For example, one Dutch study found that, compared to individuals with uncovered faces, the positive emotions of those with partially covered faces are perceived as less intense and their negative emotions as more intense, a finding that existed irrespective of whether the face-coverings involved a form of Islamic dress.^48^

### Premise 1

At this stage, some critics might say, correctly, that only small percentages of people wore the burqa, niqab, or other type of face-covering when public face-covering bans were introduced in several European countries.^49^ From this, they might infer that such bans were, and continue to be, unnecessary within these societies. On this view, it is *only once* a large proportion of the population starts publicly covering their faces that opportunities for sharing micro-moments of connection with strangers become unduly small and, accordingly, that bans on public face-covering become an acceptable means of protecting (some) people from the harmful health effects of chronic loneliness.

As indicated by premise 1, I think that this view is mistaken. Insofar as certain behaviours would cause significant harm to others’ health if enough individuals engaged in them, then this already seems to justify restricting the relevant behaviours as long as this can be done at reasonable cost *regardless of* how many individuals actually engage in them. The best way of showing this is to point to the many cases where this principle appears to hold:

*Bans on driving diesel cars in city centres:* While the emissions of a single diesel car do little harm to a city’s air quality, when many people drive such cars, this will cause significant harm. Because of this, it looks perfectly morally permissible for municipal governments to prohibit *all* diesel drivers from entering the city centre as opposed to allowing a few diesel drivers to enter.

*Smoking in pubs and bars:* Even if for a single individual to smoke in pubs and bars does not pose much of a danger to the public’s health, once millions of people do so within a country, this will create a public health hazard as pub and bar visitors, including ones who do not smoke themselves, will face significantly higher risks of developing e.g. coronary heart disease and lung cancer as a result of exposure to second-hand smoke, which seems to justify a ban on smoking within these settings for *all* visitors.

*Bans on waste-dumping:* Even if the practice of a single (small) factory to dump waste into a nearby river has a negligible impact upon the overall water quality, the fact that great environmental damage would ensue if more factories were to follow suit appears to be a sound justification for making such dumping illegal for *all* factories.

*Stay off the lawn-ordinances:* Even if the lawns in certain sections of a public park can withstand small numbers of people walking on them, the fact that they would be ruined if many people were to do so, whether synchronically or over relatively short time-intervals, seems a good enough reason to deny *everyone* access to them as opposed to simply a proportion of the park’s visitors.

In response, our critics might concede that behaviours that are collectively harmful but not individually are sometimes appropriately proscribed for all. However, they might add that this is true *only if* too many individuals would otherwise engage in them, whereby ‘too many’ means enough to produce the harms in question. Since there is no credible risk that large swathes of European populations will start publicly covering their faces once this is allowed (and it is worth noting that within those European countries where it is still allowed, this scenario has not materialised thus far), it may be concluded that bans on public face-covering are currently morally indefensible within Europe.

The problem with this argument lies in its major premise. For collectively but not individually harmful behaviours to be permissibly restricted, it does not seem necessary that failing to restrict them must cause significant harms. Consider again the waste-dumping case. Even if the practice of a single (small) factory to dump waste into a nearby river does little harm to the overall water quality, and even if social norms against waste-dumping and worries about their public reputation have the effect that other factories would not dump waste into the river even if this was legally allowed, it still looks morally permissible for the state to prohibit *all factories* from dumping waste into the river.

Our critics might agree but retort that this is *only because* dumping even a small amount of waste is likely to cause some harm to the environment, no matter how small. When this is not the case, i.e. when certain behaviours are individually entirely innocent, they might say that restricting them is justified *only if* enough people would otherwise engage in them to produce harmful outcomes. To focus attention, suppose that for a single person to walk on a park lawn or for a small number of people to do so does not do any damage to the lawn, meaning that it is *only once* a larger number starts to walk on it that the grass blades start to break and that the soil becomes compacted enough to affect the grass’ regrowth. Under these conditions, it might be argued that *unless* failing to impose restrictions upon entering the lawn will result in enough individuals entering it to damage the lawn, there should be no restrictions upon entering it.

Suppose *arguendo* that this is correct. Even then, I do not think it follows that it must be morally impermissible for contemporary European states to ban public face-covering. The reason for this is that, while for a single person to enter a park lawn might not have any negative effects, for a single person to cover her face in public has at least *some negative effects* upon other people’s opportunities to satisfy their social needs compared to a situation where the relevant individual does not publicly cover her face, assuming all else to be equal. Not only was it noted that the use of face-coverings renders communication between strangers—and, implied by this, the sharing of micro-moments of connection—much harder, we have seen that for people to cover their face will often make it (almost) impossible for those with serious hearing disabilities to have conversations with them, which is especially problematic when there are no other potential conversation partners around with uncovered faces—just think of situations where the only fellow passenger on a bus is wearing a burqa, or of ones where the only other (grand)parent at a playground is wearing a niqab. What is more, even when there are people with uncovered faces to talk to, the fact that some have covered their faces remains problematic on grounds that it still reduces the number of individuals with whom those with hearing disabilities could meaningfully interact.

### Premises 5 and 6

Thus far, I have defended four premises (1–4), which, if all true, establish that it is morally permissible for contemporary European states to ban public face-covering *as long as* this does not impose unreasonable costs or burdens (premise 5). However, I have not yet considered whether the costs and burdens of such bans are unreasonable. Some might say so on grounds that most people who wish to cover their face within these societies, namely Muslim women, seem to be motivated by deep commitments. Even if relatively few Muslims believe that the Islamic faith strictly requires women to wear a burqa or niqab, testimonial evidence suggests that a large share of burqa- and niqab-wearers within Europe regards wearing the full veil as a way of being a more exemplary Muslim and, consequently, as something that is important to their (religious) identity.^50^ For example, prior to Belgium’s face-covering ban, one woman from Brussels replied to the question of why she wore the full veil as follows: ‘Because I dress the way that I am. I already wore the veil inside of me before I started wearing it on the outside’.^51^ In a similar vein, Tahihra Noor—a British woman who has been wearing the burqa for the past 20 years—reports that, although she does not consider it to be obligatory for Muslim women to wear the burqa, she does believe that doing so ‘gets you closer to God’ by ‘emulating the Prophet Muhammad’.^52^ Further reasons for thinking that many Muslim women who wear the full veil within contemporary European societies are motivated by deep commitments come from the fact that wearing a niqab or burqa within these societies is commonly met with verbal and/or physical aggression.^53^ The assumption here is that *unless* those who wear these types of garment care deeply about this, they would normally stop wearing them to avoid such hostile responses. (While an alternative explanation would be that many of them wear the full veil because they fear being *sanctioned* by their husband and/or by other co-religionists for failing to do so, self-reports among full-veil wearing women in Denmark, Belgium, France, the United Kingdom, and the Netherlands suggest that the choice to wear the full veil is generally a voluntary one within these societies even if it is never made in a social vacuum.^54^)

What is apposite for this article’s purposes is that, if I am right that deep commitments are undergirding the wishes of a large proportion of full-veil wearing Muslims in Europe to publicly cover their face, then bans on public face-covering raise a trilemma for this group: They can (1) isolate themselves in their homes and forego many of the instrumentally and non-instrumentally valuable activities that take place in publicly accessible spaces and *only* in those spaces (e.g. attending university; working in an office; shopping; visiting parks and beaches); (2) contravene their deep commitments by appearing in public with an uncovered face; or (3) break the law by appearing in public with a covered face and risk penalties for doing so (which might complement any social criticism and abuse to which they might be exposed). Whatever they choose, the costs and/or burdens are likely to be substantial and, some will argue, unreasonably high, at least in societies where only a small minority of individuals would cover their face in the absence of face-covering bans as I suggested is the case within contemporary European societies.

While I think all of this is true, I do not believe it follows that we must oppose bans on public face-covering within these societies. The reason for this is that those with deep commitments to covering their face in public could be *exempted* from such bans in the same way that pacifists are exempted from military service in some countries.^55^ To ascertain the existence of such commitments, states might ask those seeking an exemption to submit motivation letters and/or be interviewed by a state official. If effective, this approach would ensure that no-one with deep commitments to appearing in public with a covered face is forced to show her face in public except in situations where this is necessary for purposes of identification (e.g. at airport security checks), while simultaneously ensuring that those with mere preferences for covering their face in public—perhaps in order to reduce the probability of being exposed to interactive overtures from strangers, or perhaps in order to simply experience what it is like to be fully veiled in public—would still be denied the right to do so.

What to make of this approach? Whereas it seems morally preferable to having blanket face-covering bans on grounds that it is less likely to force people to act against their religious or conscientious beliefs, I believe that it ought to be rejected in favour of a third approach. One problem with it is that requiring state officials to assess people’s motives for wanting to publicly cover their faces consumes a lot of public resources. Another is that such assessments come with a risk of error that might result in either underinclusion whereby people are denied an exemption who should have one (and who will consequently suffer unreasonable costs and/or burdens as a result being subjected to the aforementioned trilemma) or in overinclusion whereby people are granted an exemption who should not have one. Still another problem with the current approach is that it does not address the social costs of public face-covering, which it was noted are especially highly for those with hearing disabilities.

To circumvent these problems, the approach that I favour grants exemptions from face-covering bans to *anyone* who wishes to be exempted from these bans provided that they agree to perform specific services that help to protect other people from chronic loneliness. Such an approach might be compared to the alternative civilian services that countries such as Austria and Finland have introduced to accommodate citizens with conscientious or religious objections to serving in the military (e.g. Jehovah’s Witnesses, secular pacifists).^56^ While this group has the right not to serve in the military within these countries, the enjoyment of this right is conditional upon them working an x-number of months in places such as hospitals and care homes. In a similar vein, my proposal is that full-veil wearers should be able to receive exemptions from face-covering bans on the condition that they perform services that help to protect fellow members of society from chronic loneliness, for example by answering calls on anti-loneliness phone lines^57^ or by visiting lonely inhabitants of care homes, after which they would be issued a permit for publicly covering their face for a certain period that they would need to show to law enforcers upon inspection. (Of course, to satisfy the conditions of premise 6, such compensatory services must not be unduly costly or burdensome, which will require states to attend to how much time people have to perform them, as well as to whether they have any disabilities or diseases that make it difficult for them to perform (specific) services and to whether they need childcare as they perform them.)

To see how this approach improves upon the previous one, it should be noted that it does not require states to assess the sincerity and depth of people’s motives for wanting to cover their face in public, which is highly desirable as it both saves public money and avoids the risk of overinclusive and underinclusive judgements (cf. the penultimate paragraph). Another advantage that it has over the previous approach, as well as over a no-ban approach, is that by requiring exemption-seekers to perform anti-loneliness services, these individuals will offset the social costs of their covering practices as detailed earlier this section.

Lest I be misunderstood, I am not suggesting that contemporary European states *should adopt* the kind of qualified face-covering ban just outlined as opposed to having no ban at all. While I have identified some morally important reasons for doing so, my claim in this article has been more modest, namely that it is *morally permissible* for them to do so. As I have argued, even if the social costs of public face-covering are ordinarily fairly low within these societies because of the small shares of inhabitants who wear non-medical face-coverings, or who would do so if this were allowed, this is not a decisive reason against banning this type of face-covering given that its social costs *would be substantial* if larger shares were to publicly cover their face, which seems enough to justify a ban in the same way that various other behaviours that produce significant harm only at an aggregate level seem to be permissibly restricted (e.g. walking on lawns, driving diesel cars in city centres). However, if this is correct, then it looks like France and several other European countries are justified in banning non-medical face-coverings from public spaces even if they are wrong not to allow exemptions from these bans.

## Objections and Some Rejoinders

To further bolster my qualified acceptance of (non-medical) public face-covering bans, I want to defend it against four objections. The first applies to *all forms of public face-covering bans*. According to this objection, although it is true that public face-covering imposes social costs on others, especially on those with hearing disabilities, public face-coverers cannot be held morally responsible for these effects. This response is predicated on the assumptions that we lack moral duties to appear in public and that, because of this, we cannot have moral duties to appear in public *in specific ways* either, in this case without a face-cover. To the extent that laws ought to track people’s moral obligations, this would then suggest that bans on public face-covering cannot be morally justified.

I believe that this argument is unconvincing. Even if it is true that we lack moral duties to appear in public from time to time, it seems that, once we decide to leave our homes, we can be permissibly asked to abstain from dressing in ways that makes it very difficult for strangers, if not virtually impossible for some of them such as those who are deaf, to share micro-moments of connection with us provided that such abstentions do not impose unreasonable costs or burdens (see the previous section). To bring this out, notice that if moral duties to refrain from covering one’s face in public presuppose the existence of moral duties to appear in public and if such duties do not exist, then we cannot have moral duties to stay off park lawns or to stay out of city centres with our diesel cars either as these restrictions also apply to activities in public spaces. But this is clearly a *reductio ad absurdum*.^58^

A second objection says that moral duties to refrain from covering one’s face in public cannot exist because, if they did, they would be part of a broader (positive) moral duty to socially interact with strangers that would be unduly demanding. Those who raise this objection are right that simply refraining from covering one’s face in public will not result in micro-moments of connection with strangers and consequently do little to protect these individuals from chronic loneliness. For this to happen, we must also accept strangers’ interactive bids, for example by smiling back to them when they smile at us; by greeting them back when they greet us; and by engaging in small talk with them when they make conversational overtures. My response is that, *even if* we cannot always be expected to be socially responsive in these and other ways because of the burdens that this would place on us (sometimes we just want to e.g. nap in the bus or train or read a book in the park), the detrimental health effects of chronic loneliness, along with its widespread prevalence within contemporary European societies (see the previous section), makes it plausible to think that it is not too much to ask of inhabitants of these societies to engage in low-key forms of sociability like the ones just mentioned at least *some of the time*.

A third objection maintains that even qualified bans on public face-covering, although facially neutral, are morally indefensible because of their disproportionate impact on women. To see that men and women are affected unevenly by such bans, it should be noted that, while it is certainly possible for men to have desires to cover their entire face, perhaps for aesthetic reasons or because they want to reduce the risk of being subjected to social overtures by strangers, it is in most cases Muslim women who want to do so based on scriptures in the Quran that require them to dress modestly and to hide their beauty in public.^59^

The thing to say about this objection, I believe, is that, *even if* such a gender-unequal impact is morally regrettable, this is not enough to render face-covering bans unjustified within contemporary European societies. There are two reasons for this. One is that the aforementioned studies into the motives of burqa- and niqab-wearers within Europe (see the previous section) suggest that the decisions of these individuals to wear these types of clothing are generally voluntary ones even if they are never made in a social vacuum, which makes it possible to hold said individuals (largely) accountable for them. The other reason is that, by wearing the full veil, burqa- and niqab-wearers not only undermine the prospects of other persons, especially those with hearing disabilities, to have micro-moments of connection with them in public, but also help to propagate the sexist message that women but not men are to cover their beauty and to dress so modestly that they virtually become unrecognisable, which is relevant as it means that tolerating these types of dress *similarly* has a gender-unequal impact (which, to be clear, does not necessarily mean that this impact alone justifies banning them; I leave this for the reader to decide).

A fourth and final objection maintains that the sociability argument for face-covering bans proves too much. On this view, if it is morally permissible to proscribe public face-covering that involves the wearing of burqas and niqabs (i.e. what I have referred to as the ‘full veil’ for short), then in order to be consistent, states should also ban e.g. pulling a scarf over one’s face on a winter day as this renders the face (largely) invisible too, along with the wearing of medical masks and the wearing of masks during festivals such as Carnival and Halloween. Going further still, proponents of this objection might say that consistency demands that various sociability-inhibiting practices that do not involve public face-covering ought to be banned as well, such as listening to music or podcasts on trains or buses and building gated communities to which non-residents lack physical access. However, the objection continues, since such bans would unduly constrain our freedom, governments should not ban the full veil either in order to avoid imposing arbitrary—some might say discriminatory—restrictions.

I think this slippery slope-argument is unsuccessful. Upon closer inspection, there are good reasons for banning the wearing of the full veil but not most of the other sociability-inhibiting practices just mentioned:

- As for pulling a scarf over one’s head to cope with the cold, people only do this under highly specific circumstances, namely when the temperature is very low. As such, there would normally not be a justification for engaging in this practice inside shops, cafes, and pubs or on trains or buses, which is where many micro-moments of connection between strangers occur. Furthermore, being free to cover one’s face with a scarf when it is cold is something in which we *all* have interests given the human need to stay warm.
- As for the wearing of medical masks, this practice too takes place under very specific circumstances only, namely when people have an infectious disease or are at risk of having one without their knowledge. Given that we have strong societal—and often personal—interests in people masking up under these circumstances, at least when they pose a credible danger to others’ health, allowing this practice seems perfectly reasonable as well.^60^
- As for masking up for Carnival, Halloween, and other costumed festivals, there are again good reasons for making exceptions to bans on public face-covering. Apart from the fact that these types of masking happen only one evening a year (Halloween) or only a few days a year (Carnival), the joy that wearing costumes, including masks, can bring on these occasions is one that is widely accessible, including to individuals with hearing disabilities.^61^
- As for listening to music or podcasts on buses or trains, it is true that, all other things being equal, getting the attention of those engaged in this activity tends to be more difficult than getting the attention of those who are not engaged in it. Still, since at least the full veil is not readily removeable (unlike, say, a medical mask), putting on a burqa or niqab seems to create greater barriers to socialising with people with hearing disabilities than listening to music or podcasts on an audio-player, which is an activity that could be almost instantaneously ended by pressing the ‘pause-button’ or by pulling out one’s earphones.

Now, unlike the practices just mentioned, I think that there is a strong case for prohibiting the construction of gated communities when this does not put their would be-inhabitants at serious risk of being robbed and/or assaulted. The reason for this is that, compared to ungated communities, such communities make it very hard for strangers to share micro-moments of connection as non-residents lack access to the premises, apart from the fact that they help to entrench socio-economic fault lines within society. While a full defence of this claim would require a separate paper, what is pertinent for us is that, if I am right about this, then *even if* my arguments for banning public face-covering entail, or simply suggest, that gated communities ought to be banned as well, this does not count against my (qualified) defence of the former.

## Some final Clarifications

By way of conclusion, I want to end with a few clarificatory remarks. First, it bears emphasising that my defence of public face-covering has been limited to (European) countries in which chronic loneliness is rife and in which people consequently have strong interests in social environments that are conducive to micro-moments of connection between strangers. Whether such bans, or perhaps ones that simply target burqas and niqabs because of the sexist norms undergirding these types of dress, can be justified as well when (almost) everyone in society, including people with hearing disabilities, are socially well-connected is something on which I have remained non-committal. Second, nothing I have said should be taken to suggest that banning public face-covering^62^ is the *only thing* that states are morally allowed to do in order to help protect their citizens from chronic loneliness. As I have argued in other work, there is a range of other measures that may be morally permissible, if not necessary, such as educating citizens about the health hazards of this type of loneliness; ensuring that (new) hospitals and care homes can be easily reached by public transport; and loosening restrictions on family reunification.^63^ Third, especially when states introduce or maintain bans on public face-covering, it is imperative that they simultaneously address any verbal and/or physical abuse to which Muslims might be subjected, as well as that they communicate widely and clearly that these individuals are full and equal citizens insofar as this is being challenged by some groups within society (e.g. far-right nationalists).^64^ Besides being important in its own right, failing to do so risks creating the impression that their face-covering bans are motivated by Islamophobia, which could potentially render such bans unjustified. However, a discussion of this issue will have to await another occasion.

## Footnotes

1 ‘The Islamic Veil across Europe’ *BBC News* (31 May 2018). https://www.bbc.com/news/world-europe-13038095. accessed 8 September 2020.

2 E.g. Sune Lægaard, ‘Burqa Ban, Freedom of Religion and “Living Together”’ (2015) 16 Human Rights Review 203; Luara Ferracioli, ‘Challenging the Burqa Ban’ (2013) 34 Journal of Intercultural Studies 89; Shaira Nanwani, ‘The Burqa Ban: An Unreasonable Limitation on Religious Freedom or a Justifiable Restriction’ (2011) 25 Emory International Law Review 1431; Eva Brems, ‘Het “boerkaverbod” in België’ [2014] Ethische Perspectieven 3.

3 Compare Ulrike Spohn, ‘Sisters in Disagreement: The Dispute Among French Feminists About the “Burqa Ban” and the Causes of Their Disunity’ (2013) 12 Journal of Human Rights 145.

4 *S.A.S. v France* [2014] ECHR 43835/11.

5 See, for instance, Austria’s *Zivildienst.* Zivildienstserviceagentur, ‘Der Weg Zum Zivildienst’. https://www.zivildienst.gv.at/101/start.aspx. Accessed 8 September 2020.

6 Loi n° 2010–1192 du 11 octobre 2010 interdisant la dissimulation du visage dans l’espace public [Law No. 2010–1192 of 11 October 2010, Banning Concealment of the Face in Public Places].

7 Quoted in *S.A.S v France* (n 4) para 28.

8 Quoted in ibid.

9 Quoted in ibid 28–29.

10 Quoted in ibid 25.

11 This is true even though the burqa and niqab are not mentioned in the legal text.

12 Angelique Chrisafis, ‘Nicolas Sarkozy Says Islamic Veils Are Not Welcome in France’ *The Guardian* (22 June 2009). https://www.theguardian.com/world/2009/jun/22/islamic-veils-sarkozy-speech-france. Accessed 9 September 2020.

13 For a discussion, see Nanwani (n 2) 1440–1443. For the report itself, see Nationale Assemblée, ‘Rapport d’Information Au Nom de La Mission d’Information Sur La Pratique Du Port Du Voile Integral Sur Le Territoire National [Report of the Commission on the Practice of Wearing the Full Veil in France]’ (2010) 2262. http://www.assemblee-nationale.fr/13/pdf/rap-info/i2262.pdf. Accessed 13 September 2020.

14 Patrick Weil, ‘Headscarf versus Burqa: Two French Bans with Different Meanings’, *Constitutional Secularism in an Age of Religious Revival* (Oxford University Press 2014) 211–212.

15 *S.A.S v France* (n 4).

16 A ‘margin of appreciation’ refers to the amount of discretion that states have to determine whether they are complying with the European Convention of Human Rights. See Jeffrey A Brauch, ‘The Margin of Appreciation and the Jurisprudence of the European Court of Human Rights: Threat to the Rule of Law’ (2004) 11 Columbia Journal of European Law 113.

17 *S.A.S v France* (n 4) paras 157–159.

18 Wet tot instelling van een verbod op het dragen van kleding die het gezicht volledig dan wel grotendeels verbergt, 1 juni 2011. [A Law to prohibit the wearing of any clothing entirely or substantially concealing the face, 1 June 2011].

19 *Belkacemi and Oussar v Belgium* [2017] ECHR 37798/13.

20 ‘The Islamic Veil across Europe’ (n 1); Deutsche Welle, ‘Where Are “burqa Bans” in Europe?’ (*DW.COM*, 8 January 2019) < https://www.dw.com/en/where-are-burqa-bans-in-europe/a-49843292 > accessed 9 September 2020.

21 Jon Sharman, ‘Norway Votes in Favour of Banning Burqa in Schools and Universities’ *The Independent* (6 July 2018) < https://www.independent.co.uk/news/world/europe/norway-burqa-ban-schools-universities-parliament-vote-niqab-latest-a8387826.html > accessed 9 September 2020.

22 Alan Yuhas and Claire Moses, ‘Dutch Officials Decline to Enforce New Partial Ban on Burqas’ *The New York Times* (1 August 2019) < https://www.nytimes.com/2019/08/01/world/europe/netherlands-burqa-ban.html > accessed 9 September 2020.

23 *S.A.S v France* (n 4) 61.

24 Lægaard (n 2).

25 Eva Brems, ‘S.A.S. v. France as a Problematic Precedent’ (*Strasbourg Observers*, 9 July 2014) < https://strasbourgobservers.com/2014/07/09/s-a-s-v-france-as-a-problematic-precedent/ > accessed 10 September 2020.

26 Kimberley Brownlee, ‘The Right to Participate in the Life of the Society’, *Being Social: The Philosophy of Social Human Rights* (Oxford University Press Forthcoming).

27 Barbara Fredrickson, *Love 2.0: Finding Happiness and Health in Moments of Connection* (Penguin 2013).

28 ibid 15.

29 Bouke De Vries, ‘Four Types of Anti-Loneliness Policies’ in Kimberley Brownlee, David Jenkens, and Adam Neal (eds), *Being Social: The Philosophy of Social Human Rights* (Oxford University Press Forthcoming).

30 Jenny De Jong Gierveld and Tineke Fokkema, ‘Strategies to Prevent Loneliness’ in Ami Sha’ked and Ami Rokach (eds), *Addressing Loneliness: Coping, Prevention and Clinical Interventions* (1 edition, Psychology Press 2015).

31 John T Cacioppo, Louise C Hawkley, and Ronald A Thisted, ‘Perceived Social Isolation Makes Me Sad: Five Year Cross-Lagged Analyses of Loneliness and Depressive Symptomatology in the Chicago Health, Aging, and Social Relations Study’ (2010) 25 Psychology and Aging 453.

32 Tjalling Jan Holwerda and others, ‘Feelings of Loneliness, but Not Social Isolation, Predict Dementia Onset: Results from the Amsterdam Study of the Elderly (AMSTEL)’ (2014) 85 Journal of Neurology, Neurosurgery, and Psychiatry 135.

33 Ingemar Åkerlind and Jan O Hörnquist, ‘Loneliness and Alcohol Abuse: A Review of Evidences of an Interplay’ (1992) 34 Social Science & Medicine 405.

34 Ye Luo and others, ‘Loneliness, Health, and Mortality in Old Age: A National Longitudinal Study’ (2012) 74 Social science & medicine (1982) 907.

35 Mette M Aanes, Maurice B Mittelmark, and Jørn Hetland, ‘Interpersonal Stress and Poor Health: The Mediating Role of Loneliness’ (2010) 15 European Psychologist 3.

36 Amny Novotney, ‘The Risks of Social Isolation’ (*APA*, May 2019) < https://www.apa.org/monitor/2019/05/ce-corner-isolation > accessed 13 September 2020.

37 Fredrickson (n 28) 51–52.

38 Vivid evidence of this is provided by individuals who come to their General Practioner’s surgery because they are lonely, a phenomenon that is reported to occur 1–5 times a day by 76 percent of British GPs. See Campaign to End Loneliness, ‘Family Doctors Ill-Equipped for Loneliness Epidemic’ (2013) < https://www.campaigntoendloneliness.org/wp-content/uploads/downloads/2013/11/FINAL-GP-Polling-PR-15.11.13.pdf > accessed 13 September 2020.

39 Frieder R Lang and Laura L Carstensen, ‘Close Emotional Relationships in Late Life: Further Support for Proactive Aging in the Social Domain’ (1994) 9 Psychology and Aging 315.

40 Pearl A Dykstra, ‘Older Adult Loneliness: Myths and Realities’ (2009) 6 European Journal of Ageing < https://www.ncbi.nlm.nih.gov/pmc/articles/PMC2693783/ > accessed 26 June 2020.

41 TG van Tilburg, ‘Typen van eenzaamheid’ in TG van Tilburg and J de Jong-Gierveld (eds), *Zicht op eenzaamheid: Achtergronden, oorzaken en aanpak* (Van Gorcum 2007) 33 < https://research.vu.nl/en/publications/zicht-op-eenzaamheid-achtergronden-oorzaken-en-aanpak > accessed 30 September 2018.

42 ibid; Christina R Victor and Keming Yang, ‘The Prevalence of Loneliness among Adults: A Case Study of the United Kingdom’ (2012) 146 The Journal of Psychology 85.

43 World Health Organization, ‘Deafness and Hearing Loss’ (3 January 2020) < https://www.who.int/news-room/fact-sheets/detail/deafness-and-hearing-loss > accessed 11 September 2020.

44 This is true when the use of sign language is not an option, as it frequently is not, given that it is quite rare for random strangers to know (the local) sign language. Furthermore, even sign languages do not rely exclusively upon manual signs but make heavy use of facial expression as well. See Eeva A Elliott and Arthur M Jacobs, ‘Facial Expressions, Emotions, and Sign Languages’ (2013) 4 Frontiers in Psychology 2 < https://www.ncbi.nlm.nih.gov/pmc/articles/PMC3593340/ > accessed 11 September 2020.

45 Jasmine Taylor-Coleman, ‘Call for Clear Face Masks to Be “the Norm”’ *BBC News* (26 May 2020) < https://www.bbc.com/news/world-52764355 > accessed 11 September 2020.

46 ibid.

47 Carlos Crivelli and Alan J Fridlund, ‘Facial Displays Are Tools for Social Influence’ (2018) 22 Trends in Cognitive Sciences 388.

48 Agneta H Fischer and others, ‘Veiled Emotions: The Effect of Covered Faces on Emotion Perception and Attitudes’ (2012) 3 Social Psychological and Personality Science 266.

49 At the time that France introduced its face-covering ban, it was estimated that approximately 1,900 women wore the full veil out of a population of approximately 64 million. See Nanwani (n 2) 1437. At the time that Belgium introduced its face-covering ban, estimates were that between 200 and 270 women wore the full veil out of a population of circa 11 million. See Brems (n 2) 19.

50 See e.g. Eva Brems, *The Experiences of Face Veil Wearers in Europe and the Law* (Cambridge University Press 2014); Brems (n 2).

51 ‘Parce que c’est mon comportement, pour moi si vous voulez, mon voile je l’ai d’abord porté à l’intérieur avant de le mettre à l’extérieur’. Quoted in Brems (n 2) 7.

52 BBC, ‘Why Some Muslim Women Wear the Veil’ *BBC News* (8 August 2018). https://www.bbc.com/news/uk-45112792 > accessed 20 September 2020.

53 Brems (n 2) 16.

54 See e.g. Nanwani (n 2); Brems (n 2); Brems (n 50).

55 Compare Yossi Nehushtan, ‘The Case for a General Constitutional Right to Be Granted Conscientious Exemption’ (2016) 5 Oxford Journal of Law and Religion 230.

56 See Zivildienstserviceagentur (n 5).

57 For an example of such a phone line, see Rachel Looker, ‘Phone Line Reduces Senior Loneliness’ (*NACo*, 12 September 2019) < https://www.naco.org/articles/phone-line-reduces-senior-loneliness > accessed 12 September 2020.

58 One might also consider a case discussed by philosopher Shelley Kagan where a person who is a bird fanatic goes into a burning building and can either save a parrot or a young child. Despite the fact that the person might not have a moral duty to go into the burning building, once he does so, most people would say that he has a moral duty to save the child rather than the parrot. See Shelly Kagan, *The Limits of Morality* (Clarendon Press 1991).

59 I am indebted to an anonymous referee for raising this objection.

60 Indeed, rather than contravening the ideal of ‘living together’ as wearing a burqa or niqab does, wearing such facemasks may be plausibly said to be a *requirement* of this ideal as it shows concern for other people’s health.

61 Notice further that, whereas wearing face-covering costumes during Carnival or Halloween might complicate social interactions with strangers in one respect by rendering it more difficult to read people’s lips and faces, such costumes simultaneously provide easy conversation topics in addition to contributing to a festive atmosphere in which people may be more likely to strike up conservations with strangers, which is why some might resist calling the wearing of such costumes a ‘sociability-inhibiting practice’.

62 Subject to the provisions discussed in the previous section.

63 De Vries (n 29).

64 Compare Corey Brettschneider, *When the State Speaks, What Should It Say?: How Democracies Can Protect Expression and Promote Equality* (Princeton University Press 2012).
